# Responses of Parasitoids to Volatiles Induced by *Chilo partellus* Oviposition on Teosinte, a Wild Ancestor of Maize

**DOI:** 10.1007/s10886-015-0570-1

**Published:** 2015-05-06

**Authors:** Daniel M. Mutyambai, Toby J. A. Bruce, Charles A. O. Midega, Christine M. Woodcock, John C. Caulfield, Johnnie Van Den Berg, John A. Pickett, Zeyaur R. Khan

**Affiliations:** Habitat Management Programme, International Centre of Insect Physiology and Ecology, P.O Box 30-40305, Mbita, Kenya; Department of Biological Chemistry, Rothamsted Research, Harpenden, Herts AL5 2JQ UK; Unit for Environmental Sciences and Management, North-West University, Potchefstroom, 2520 South Africa; Zoological Services, Veterinary Department, Ministry of Agriculture, Livestock and Fisheries, P.O. Box 30028-00100, Nairobi, Kenya

**Keywords:** Induced defense, oviposition, plant volatiles, tritrophic interactions, crop wild relative

## Abstract

Maize, a genetically diverse crop, is the domesticated descendent of its wild ancestor, teosinte. Recently, we have shown that certain maize landraces possess a valuable indirect defense trait not present in commercial hybrids. Plants of these landraces release herbivore-induced plant volatiles (HIPVs) that attract both egg [*Trichogramma bournieri* Pintureau & Babault (Hymenoptera: Trichogrammatidae)] and larval [*Cotesia sesamiae* Cameron (Hymenoptera: Braconidae)] parasitoids in response to stemborer egg deposition. In this study, we tested whether this trait also exists in the germplasm of wild *Zea* species. Headspace samples were collected from plants exposed to egg deposition by *Chilo partellus* Swinhoe (Lepidoptera: Crambidae) moths and unexposed control plants. Four-arm olfactometer bioassays with parasitic wasps, *T. bournieri* and *C. sesamiae*, indicated that both egg and larval parasitoids preferred HIPVs from plants with eggs in four of the five teosinte species sampled. Headspace samples from oviposited plants released higher amounts of EAG-active compounds such as (*E*)-4,8-dimethyl-1,3,7-nonatriene. In oviposition choice bioassays, plants without eggs were significantly preferred for subsequent oviposition by moths compared to plants with prior oviposition. These results suggest that this induced indirect defence trait is not limited to landraces but occurs in wild *Zea* species and appears to be an ancestral trait. Hence, these species possess a valuable trait that could be introgressed into domesticated maize lines to provide indirect defense mechanisms against stemborers.

## Introduction

Emission of herbivore-induced volatiles (HIPVs) in response to herbivore feeding damage has been studied intensively during the past two decades. Recently, a relatively small but growing number of investigations have revealed that oviposition can induce production of plant volatiles that attract parasitoids (Bruce et al. 2010; Colazza et al. 2004; Fatouros et al. 2012; Hilker and Meiners 2006; Tamiru et al. 2011, 2012). Detection of egg deposition and subsequent changes in chemical phenotype prepares plants for the impending attack by emerging phytophagous larvae (Bruce et al. 2010; Hilker et al. 2002). Natural enemies of herbivores make use of these HIPVs for host location (Dicke and Sabelis 1988; Powell, et al. 1998; Turlings et al. 1990), but different plant species release entirely different HIPV blends, and, even within one plant species, there are clear differences among genotypes (Gouinguené et al. 2001; Takabayashi et al. 1991; Turlings et al. 1998).

Information regarding variability in HIPV emission comes mainly from studies of cultivated plants (Gouinguené et al. 2001; Krips 2000; Takabayashi et al. 1991; Turlings et al. 1998). Studies of wild systems include a wild cotton variety that was found to release much higher quantities of induced volatiles than cultivated varieties (Loughrin et al. 1995), and of larval regurgitant applied to mechanically damaged maize leaves (Gouinguené et al. 2001). However, no studies of tritrophic interactions have yet investigated wild maize following egg deposition by a herbivore. For a better understanding of the ecological relevance and evolutionary history of oviposition-induced plant signalling, it is necessary to study these signals in wild systems.

The spotted stemborer, *Chilo partellus* (Swinhoe) (Lepidoptera: Crambidae) is one of the most damaging lepidopteran pests of maize in eastern and southern Africa and Asia, causing yield losses of up to 88 % (Kfir et al. 2002). Effective chemical control is difficult, mostly due to the protection provided to larvae by plants when feeding inside plant whorls and stems (Slabbert and Van den Berg 2009). Furthermore, insecticides are not economical for smallholder farmers. Thus, the ecology of tritrophic interactions presents an opportunity for development of crop protection approaches that make use of induced innate plant defenses.

For better understanding and utilization of indirect defense traits in pest management strategies, we need to consider their evolutionary and ecological history when subjected to forces such as domestication or breeding. A recent review states that further research into pest resistance of maize wild ancestors, the teosintes, is needed (de Lange et al. 2014). The objectives of this study were, therefore, to determine: (1) volatile profile changes in response to *C. partellus* egg deposition on wild maize; (2) behavioral responses of parasitoids to HIPVs collected from wild maize exposed to stemborer oviposition; and (3) the effect of oviposition-induced volatiles on subsequent moth oviposition on these plants.

## Methods and Materials

### Plants

Seeds of the following teosinte species were obtained from the International Maize and Wheat Improvement Centre (CIMMYT), Mexico: *Zea diploperennis* Iltis, Doebley & Guzman, *Z. huehuetenangensis* (Iltis & Doebley) Doebley, *Z. mays* spp. *mexicana* (Schrader) Iltis, *Z. m.* spp. *parviglumis* Iltis & Doebley, and *Z. perennis* (Hitcht.) Reeves & Manglesdorf. Seeds were grown individually in pots filled with fertilized soil in an insect-proof screen house at the *icipe*-Thomas Odhiambo campus, Mbita point (0° 25′S, 34° 12′E; 1200 m above sea level), in Western Kenya. All seedlings were grown under natural conditions (25 °C, 65%RH; 12L: 12D) and used in the experiments when 3–4 wk-old.

### Insects

*Chilo partellus* moths were obtained from the insect mass rearing unit at the *icipe*-Thomas Odhiambo campus. The larvae originated from field-collected stemborers, principally from sorghum (*Sorghum bicolor* L. Moench) fields in the Mbita region. Larvae were reared on a semi-synthetic diet containing sorghum leaf powder (Ochieng et al. 1985). Field collected egg parasitoids, *Trichogramma bournieri* Pintureau & Babault (Hymenoptera: Trichogrammatidae) and larval parasitoids, *Cotesia sesamiae* Cameron (Hymenoptera: Braconidae), were reared on stemborer eggs and larvae, respectively, using methodologies described by Overholt et al. (1994). The experimental insects were maintained at 24 ± 3 °C, 70 ± 5 % RH, 12L: 12D.

### Chemicals

Authentic chemical standards (>95 % purity) of (*E*)-2-hexenal, (*Z*)-3-hexen-1-ol, (*Z*)-3-hexenyl acetate, (*E*,*E*)-2,4-heptadienal, (*Z*)-2-heptenal, hexanal, decanal, nonane, α-pinene, 2,3-butanediol, myrcene, limonene, 6-methyl-5-hepten-2-one, 3,4-dimethylacetophenone, and (*E*,*E*)-farnesyl chloride were purchased from Sigma Aldrich (Gillingham, UK). (*E*)-β-Farnesene (99 % pure by GC) was synthesized from (*E*,*E*)-farnesyl chloride (Kang et al. 1987). (*E*)-4,8-Dimethyl-1,3,7-nonatriene (>98 %) was synthesised from geraniol by oxidation to the aldehyde followed by Wittig methylenation (Leopold 1990).

### Volatile Organic Compound (VOC) Collection

Headspace sampling (Agelopoulos et al. 1999) was used to collect volatile compounds from whole maize plants, with and without stemborer eggs. Prior to volatile collection, seedlings were placed in oviposition cages (80 × 40 × 40 cm) into which five gravid naïve female stemborer moths were introduced and kept overnight for oviposition. A wad of cotton wool (10 cm diam) moistened with water was placed into the cage for moths to feed on. Control plants were kept inside similar cages but without *C. partellus* moths. Volatiles were collected from these plants for a period of 48 h, starting at the last 2 h of the photophase of the following day. Leaves with or without eggs were enclosed in polyethyleneterephthalate (PET) bags (3.2 L, ~ 12.5 mm thickness) heated to 150 °C before use, and fitted with a swagelock inlet and outlet ports. Charcoal-filtered air was pumped (600 ml min^−1^) through the inlet port. Volatiles were collected on Porapak Q (0.05 g, 60/80 mesh; Supelco) filters inserted into the outlet through which air was drawn at 400 ml min^−1^. Elution of the entrained volatiles was done using 0.5 ml dichloromethane. The eluted samples were stored in tightly capped microvials in a −20 °C freezer prior to bioassays and further analysis. Entrainments from both oviposited and control plants were replicated four times, and each plant was used only once.

### Four-arm Olfactometer Bioassay

Responses of parasitoids to plant derived volatiles were tested in a Perspex four-arm olfactometer (Pettersson 1970). A choice-test was carried out to compare insect responses to headspace samples from oviposited and control plants. Two opposing arms held the test stimuli (10 μl aliquots of headspace sample). The remaining two arms held solvent controls. Olfactometers were used only once for each replicate of the bioassay, and they were washed and air dried before the next test. The experiment was replicated 12 times. Headspace samples (10 μl aliquots) were applied, using a micropipette (Drummond ‘microcap’, Drummond Scientific Co., Broomall, PA, USA), to a piece of filter paper (4 × 25 mm) placed in the inlet port at the end of each olfactometer arm. To avoid bias, the position of the treatments was randomly re-allocated between each tests, and the olfactometer was rotated after every 4 min during the test. One-day-old gravid female parasitoids without any prior exposure to plants or hosts were isolated from the rearing cage and allowed to acclimatize to room temperature for 1 h. They were transferred individually into the central chamber of the olfactometer using a custom-made piece of glass tubing. Air was drawn through the four arms towards the centre at 260 ml min^−1^. The time spent by parasitoids in each olfactometer arm was recorded with ‘Olfa’ software (F. Nazzi, Udine, Italy) for 12 min.

### Gas Chromatography (GC) Analysis

Entrained VOCs were analyzed using a Hewlett-Packard 7890 GC machine (Agilent Technologies) equipped with a cool-on column injector, a non-polar HP-1 capillary column (50 m, 0.32 mm internal diam, 0.52 μm film thickness) and a flame ionization detector (FID). Four μl of headspace sample were injected into the injector port of the GC instrument. The oven temperature was maintained at 30 °C for 2 min and then programmed at 5 °C min-1 to 250 °C. The carrier gas was hydrogen. Data were analyzed using HP Chemstation software. Quantification was done by dividing peak areas by known amounts of external standards. The emission rate in terms of ng plant^−1^ h^−1^ was obtained by multiplying inverse of the proportion of the total headspace used and dividing by the number of hours in the sampling period. Four replicate headspace samples were analyzed for each treatment.

### Coupled GC-Electroantennography (GC-EAG) Analysis

GC-EAG was carried out using the antennae of female *C. sesamiae* with the headspace samples of the teosinte species that had elicited positive responses during olfactometer bioassays. The GC-EAG system, in which the effluent from the GC column is simultaneously directed to the antennal preparation and the GC detector, was described previously by Wadhams (1990). EAG recordings were made using Ag-AgCl glass electrodes filled with saline solution, as described by Maddrell (1969) but without glucose. A female parasitoid was chilled for 1 min, and the head was excised, and the tips of both antennae were removed to ensure a good contact in the recording electrode. The indifferent electrode was placed within the head capsule. Signals were passed through a high impedance amplifier (UN-06; Syntech, Hilversum, The Netherlands) and analyzed using a customized Syntech software package. Separation of volatiles was done on a 6890 N GC (Agilent Technologies) equipped with a cold on-column injector and a FID using an HP-1 column (50 m, 0.32 mm internal diam, 0.52 μm film thickness). The oven temperature was maintained at 30 °C for 2 min and then programmed at 15 °C min^−1^ to 250 °C. The carrier gas was helium. Outputs from the EAG amplifier and the FID were analyzed using Syntech software.

### Coupled GC-Mass Spectrometry (GC-MS) Analysis

Aliquots of attractive headspace samples were analyzed using an Hewlett-Packard 5890 GC machine (Agilent Technologies) equipped with an HP-1 column (50 m, 0.32 mm internal diam, 0.52 μm film thickness) directly coupled to a mass spectrometer (VG Autospec; Fisons Instruments, Manchester, UK) equipped with a cool on-column injector. Ionization was performed by electron impact (70 eV at 250 °C). The oven temperature was maintained at 30 °C for 5 min and then programmed at 5 °C min^−1^ to 250 °C. Tentative identifications were made by comparison of mass spectra with those collected in mass spectral databases (NIST 2005). Tentative identifications of the compounds were confirmed through co-injections with authentic standards.

### Oviposition Bioassay

Two-choice tests were conducted using modifications of the methodology of Khan et al. (2007) in oviposition cages (80 × 40 × 40 cm) covered by fine cloth mesh netting with a cloth access flap. Prior to the two-choice test, a 3–4-wk-old potted maize plant was caged overnight with five gravid naïve *C. partellus* moths to allow oviposition. A wad of cotton wool (10 cm diam) moistened with water was introduced into the cage for the moths to feed on. After 24 h, the positions of egg batches on leaves were marked. The following day, another maize plant of the same species and age but without prior exposure to moths was placed into each of the oviposition cages, adjacent to the previously exposed plant. Thus, each cage had two potted maize plants positioned at opposite sides, one exposed to moths the day before, and the other without any prior exposure to moths. Five new gravid naïve *C. partellus* moths then were introduced into the cage and allowed to oviposit for 48 h under natural conditions of approximately L12:D12. Plants were removed, and the number of newly laid eggs and egg batches on each plant were counted under a light binocular microscope. ‘Preference’ was taken in this context to be differential oviposition on a plant when the insect is given a choice between two plants of the same variety but with different treatments. Data collected were expressed as the mean proportion (percentage) of total number of eggs oviposited during the second oviposition period on plants in the two-choice test. This experiment was replicated 10 times.

### Statistical Analyses

Statistical analyses were done using R software (version 3.0.2). Time spent in each arm of the four-arm olfactometer bioassay was compared by analysis of variance (ANOVA) after conversion of the data into proportions of the total time the insect was allowed to make its choice (12 min), followed by a log ratio transformation to allow analysis of compositional data (Aitchison 1981; Tamiru et al. 2011). Means were separated using the Tukey test with α set at 0.05. The two-sample (unpaired) student’s *t*-test was used to determine if there were any differences between the numbers of eggs and egg batches laid on plants that were previously either exposed or non-exposed to oviposition.

## Results

### Behavioral Responses of Parasitoids to Headspace Samples of VOCs

**Fig. 1:**
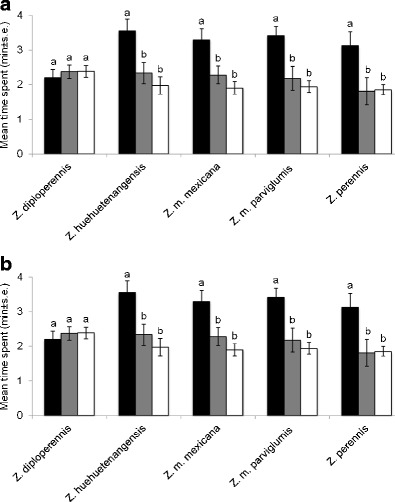
Behavioral response of female parasitoids to volatiles collected from teosinte with *C. partellus* eggs (exposed), without *Chilo partellus* eggs (non-exposed) and solvent control in a four-arm olfactometer bioassay. (**a**) response of *Trichogramma bournieri*; (**b**) response of *Cotesia sesamiae*. Each female parasitoid was observed for 12 min (*N* = 12). *Bars* followed by *different letters* are significantly different at *P* < 0.01

Female *T. bournieri* individuals spent significantly more time in olfactometer arms containing volatiles from plants exposed to oviposition in comparison to those with volatiles from plants without eggs and solvent controls, for four of the five teosinte species (*Z. huehuetenangensis F*_2,33_ = 6.505, *P* = 0.004; *Z. m.* spp. *mexicana F*_2,33_ = 4.41, *P* = 0.020; *Z. m.* spp. *parviglumis F*_2,33_ = 7.357, *P* = 0.002; and *Z. perennis F*_2,33_ = 6.492, *P* = 0.004) (Fig. 1a). Similar results were observed for larval parasitoids, *Cotesia sesamiae* (*Z. huehuetenangensis F*_2,33_ = 8.428, *P* = 0.001; *Z. m.* spp. *mexicana F*_2,33_ = 10.15, *P* < 0.001; *Z. m.* spp. *parviglumis F*_2,33_ = 10.15, *P* < 0.001; *Z. perennis F*_2,33_ = 5.488, *P* = 0.009) (Fig. 1b). In the case of *Z. diploperennis*, there were no significant differences in time spent in arms with volatiles from plants exposed to egg deposition, plants without eggs and solvent controls for both egg and larval parasitoids (*F*_2_,_33_ = 0.434, *P* = 0.651; *F*_2,33_ = 0.391, *P* = 0.679, respectively).

### Comparison of Volatiles Emitted from Plants with and Without Eggs

GC analysis revealed quantitative and qualitative changes in the volatile blend profile emitted by plants exposed to egg deposition (Table 1). *Zea mays parviglumis*, *Zea mays mexicana*, and *Zea perennis* emitted more EAG active compounds when exposed to *C. partellus* oviposition compared to unexposed plants.

**Table 1:** Volatile emission (ng / plant / h) (mean ± s.e.) from teosinte species with and without *Chilo partellus* eggs (*N* = 4)

	*Zea mays parviglumis*		*Zea mays mexicana*		*Zea huehuetenangensis*		*Zea perennis*		*Zea diploperennis*	
	With eggs	No eggs	With eggs	No eggs	With eggs	No eggs	With eggs	No eggs	With eggs	No eggs
(*E*)-2-hexenal	0.012 (±0.012)	n.d.	n.d.	n.d.	n.d.	n.d.	0.005 (±0.005)	n.d.	n.d.	n.d.
(*Z*)-3-hexen-1-ol	0.014 (±0.014)	n.d. n.d.	n.d.	n.d.	n.d.	n.d.	0.004 (±0.004)	n.d.	n.d.	n.d.
nonane	0.004 (±0.004)	n.d. n.d.	n.d.	n.d.	n.d.	n.d.	0.001 (±0.001)	n.d.	n.d.	n.d.
α-pinene	0.075 (±0.074)	0.007 (±0.007)	n.d.	n.d.	n.d.	n.d.	0.020 (±0.020)	0.001 (±0.001)	n.d.	0.002 (±0.002)
6-methyl-5-hepten-2-one	0.073 (±0.071)	0.005 (±0.005)	n.d.	n.d.	n.d.	n.d.	0.013 (±0.012)	0.002 (±0.002)	0.003 (±0.003)	n.d.
(*Z*)-3-hexenyl acetate	0.039 (±0.033)	0.021 (±0.010)	0.042 (±0.019)	0.013 (±0.005)	0.031 (±0.004)	0.013 (±0.006)	0.026 (±0.017)	0.009 (±0.009)	0.036 (±0.008)	0.008 (±0.008)
limonene	0.020 (±0.020)	0.009 (±0.009)	0.003 (±0.003)	n.d.	0.014 (±0.014)	0.007 (±0.004)	0.016 (±0.016)	0.003 (±0.003)	n.d.	0.021 (±0.021)
(*E*)-4,8-dimethyl-1,3,7 nonatriene (DMNT)	0.169 (±0.128)	0.065 (±0.029)	0.043 (±0.043)	n.d.	0.029 (±0.011)	0.010 (±0.007)	0.012 (±0.009)	0.014 (±0.009)	n.d.	0.036 (±0.021)
decanal	0.008 (±0.008)	0.006 (±0.006)	0.016 (±0.016)	n.d.	n.d.	n.d.	0.053 (±0.047)	0.009 (±0.009)	n.d.	0.045 (±0.045)
3,4-dimethylacetophenone	0.017 (±0.017)	0.019 (±0.009)	0.064 (±0.045)	n.d.	0.026 (±0.005)	0.008 (±0.006)	0.013 (±0.011)	n.d.	n.d.	n.d.
(*E*)-β-farnesene	0.012 (±0.008)	0.002 (±0.002)	0.003 (±0.003)	n.d.	0.055 (±0.041)	0.024 (±0.014)	0.026 (±0.026)	0.004 (±0.004)	0.008 (±0.008)	n.d.

*n.d.* not detected

### Identification of Attractive Volatile Organic Compounds

**Fig. 2:**
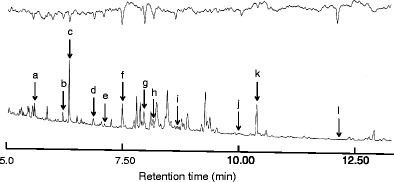
A representative GC-EAG response of female *Cotesia sesamiae* to volatiles collected from *Zea perennis* with eggs. FID peaks marked are those which elicited antennal response in coupled runs: a = hexanal, b = 2,3-butanediol, c = (*E*)-2-hexenal, d = (*Z*)-3-hexen-1-ol, e = nonane, f = (*Z*)-2-heptenal, g = 6-methyl-5-heptene-2-one, h = (*E,E*)-2,4-heptadienal, i = limonene, j = (*E*)-4,8-dimethyl-1,3,7-nonatriene (DMNT), k = decanal, l = (*E*)-β-farnesene

GC-EAG recordings with the attractive samples from teosinte species and subsequent GC-MS identification showed that *C. sesamiae* antennae were responsive to hexanal, 2,3-butanediol, (*E*)-2-hexenal, (*Z*)-3-hexen-1-ol, nonane, (*Z*)-2-heptenal, 6-methyl-5-heptene-2-one, (*E*,*E*)-2,4-heptadienal, limonene, (*E*)-4,8-dimethyl-1,3,7-nonatriene (DMNT), decanal, and (*E*)-β-farnesene (Fig. 2).

### Oviposition Preference

In two-choice tests, a significantly higher number of eggs was laid on unexposed control plants in comparison to plants with prior egg deposition, except for *Z. diploperennis* (Table 2). Similarly, a higher number of egg batches and number of eggs per batch were laid in *Z. huehuetenangensis, Z. mays* spp. *mexicana*, *Z. m.* spp. *parviglumis,* and *Z. perennis,* although the difference was only significant in *Z. m.* spp. *parviglumis* (*P* = 0.04) (Table 2).

**Table 2:** Percentages of *Chilo partellus* eggs laid per plant (± SEM), number of egg batches per plant (± SEM), and number of eggs per egg batch (± SEM) for five teosinte species exposed and unexposed to prior egg deposition

Teosinte species: oviposited (T) vs. unoviposited (C)	Mean % of eggs per plant	Mean number of egg batches per plant	Mean number of eggs per batch
*Zea diploperennis* (T)	50.79 (±9.17) a	2.83 (±0.65) a	25.92 (±4.42) a
*Z. diploperennis* (C)	49.21 (±9.17) a	3.00 (±0.52) a	25.10 (±8.93) a
*Z. huehuetenangensis* (T)	45.99 (±2.65) a	9.89 (±0.98) a	34.62 (±3.08) a
*Z. huehuetenangensis* (C)	54.01 (±2.65) b	10.33 (±0.73) a	36.62 (±5.58) a
*Z. mays* spp. *mexicana* (T)	37.43 (±6.10) a	3.90 (±0.98) a	30.72 (±4.76) a
*Z. mays* spp. *mexicana* (C)	62.57 (±6.10) b	6.40 (±0.86) a	38.92 (±8.90) a
*Z. m.* spp. *parviglumis* (T)	36.46 (±6.83) a	4.50 (±1.07) a	29.60 (±2.96) a
*Z. m.* spp. *parviglumis* (C)	63.54 (±6.83) b	7.63 (±1.31) a	37.02 (±6.32) b
*Z. perennis* (T)	34.10 (±6.79) a	10.17 (±2.40) a	20.30 (±2.76) a
*Z. perennis* (C)	65.90 (±6.70) b	13.00 (±2.73) a	32.77 (±5.90) a

Values in a column (between each oviposited and unoviposited teosinte species), followed by the same letter, are not significantly different at *P* = 0.05 (two-sample *t*-test)

## Discussion

Our findings provide evidence that indirect defense involving insect egg-induced HIPV emission is an ancestral trait in maize, present in several species of teosinte. Our previous study (Tamiru et al. 2011) showed that the egg-induced HIPV emission trait was present in certain landraces, but not in the commercial hybrid maize varieties tested to date.

Within the teosinte species, there was variation in the quality and quantity of volatiles emitted following egg deposition. Noticeably, *Z. m.* spp. *parviglumis*, which is considered the closest relative to cultivated maize (Doebley and Wang 1997; Kellogg 1997), produced more HIPVs after egg deposition, whereas *Z. diploperennis* showed little change in volatile emission. (*E*)-4,8-Dimethyl-1,3,7-nonatriene (DMNT), a key semiochemical known to attract *C. sesamiae* larval parasitoids (Khan et al. 1997), was released in larger amounts in three of the teosinte species exposed to egg deposition: *Z. m.* spp. *mexicana*, *Z. m.* spp. *parviglumis,* and *Z. perennis.* There was some overlap in the blends of EAG active egg induced volatiles emitted by teosinte species and the maize landraces we studied previously (Tamiru et al. 2011, 2012), with compounds such as DMNT, decanal, and (*E*)-β-farnesene being emitted by both. However, there were also differences, for example, methyl salicylate and TMTT were more important in the landraces, and 6-methyl-5-heptene-2-one occurred in the teosinte species. Interspecific as well as intraspecific variation in HIPV emission following larval damage of different maize breeding lines, as well as teosinte varieties, has been reported (Degen et al. 2004; Gouinguené et al. 2001), However, egg induced effects have not previously been investigated in wild maize species.

In behavioral bioassays, in four of the five species tested, both egg and larval parasitoids preferred volatiles from plants exposed to egg deposition compared to those from unexposed plants. The only species for which this effect was not observed was *Z. diploperennis*, the teosinte species that showed no induction of volatiles after exposure to oviposition and possibly a decrease. The attraction of both egg and larval parasitoids is considered a preventive defense strategy since larval parasitoids are recruited in advance, before the phytophagous larvae emerge from eggs and start causing plant damage (Bruce et al. 2010). Conversely, the herbivore itself avoided egg exposed plants. Gravid *C. partellus* moths preferred to oviposit on teosinte plants with no prior exposure to egg deposition in the four of the five species for which a change in HIPV profile was observed. However, there was no preferential oviposition behavior observed for *Z. diploperennis*. We hypothesize that the presence of volatiles attractive to the herbivore’s natural enemies could have influenced oviposition behavior, or that moths could have used this as a mechanism to avoid intraspecific competition. Previous studies have shown that HIPVs may indicate pressure from natural enemies and the risk of competition for resources on plants emitting HIPVs, resulting in female moths avoiding these plants (Dicke and Baldwin 2010; Heil and Karban 2010; Zakir et al. 2013).

The current results showed that oviposition-induced plant signalling is an ancestral trait in maize that exists even in pre-domestication germplasm. This valuable indirect defense trait is, therefore, not limited to the *Zea mays* landraces in which we originally discovered the trait (Tamiru et al. 2011). The observation of egg-induced HIPV emission in wild *Zea* species and landraces, but not in the commercial hybrids investigated to date, suggests that breeding for yield and palatability could have resulted in the loss of secondary defense metabolites in improved maize breeding lines (Benrey et al. 1998). The latter were developed primarily with the aim of higher yield and improved grain quality. In hybrid breeding programmes, pesticide applications often are used to protect breeding lines, which may have resulted in elite maize breeding material losing some of its natural defense traits. However, smallholder farmers in Africa often do not have access to insecticides, which means that when crops are grown under unprotected conditions, the loss of natural plant resistance traits, such as the indirect defense trait described here, can lead to yield instability due to crop losses caused by insect pests.
